# Letter from A. W. Bosworth, Paris

**Published:** 1870-06

**Authors:** A. W. Bosworth

**Affiliations:** Paris


### Foreign Correspondence.

Paris, April 22nd.

During the last few months there has been an epidemic of small-pox, in Paris, of considerable gravity.

The following table indicates the number of deaths from this disease in the hospitals and in private life included:

| | 1868. | 1869. | 1870. |
|---|---|---|---|
| January, | 80 | 64 | 183 |
| February, | 71 | 49 | 302 |
| March, | 76 | 62 | 411 |

The mortality has been in proportion to the wealth and poverty of the different quarters of the city—the poorer the quarter, the greater the number of deaths.

The greater number of cases of the disease has been observed to be with adults, especially between the ages of 20 and 30 years. This latter fact is explained for Paris by the census showing that there are five times as many adults in Paris as children, and four times as many old people as children. A larger number of males than of females have been attacked; yet, comparatively, more of the feminine sex have succumbed than of the former.

This epidemy has also clearly shown that the mortality is very great when attacking children during the first year of their existence—ten times greater than between the ages of 20 and 30. It has been especially very destructive to children under three months of age—thus showing that vaccination ought always to be done as near birth as possible.

The Medical Commission, appointed by the hospitals of Paris, in their report, said, in regard to the effects of vaccination: "We all know that in a certain number of cases its benefit disappears to such a degree, that certain persons having beautiful vaccinal cicatrices succumb from confluent variola, or more often from the malignant, and especially the hemorrhagic forms; we know that a larger number of vaccinated persons are attacked with variola discreta, which sometimes present great gravity, and may terminate by death; but we also know that these facts are only the exception, and that the majority of vaccinated persons are only lightly attacked; and, on the contrary, we are not ignorant if it happens that persons not vaccinated are slightly attacked, this is by far the greater exception; and, that for persons not vaccinated, when attacked with small-pox, the danger, the confluence and the malignity constitute the rule and not the exception." The Commission here give statistics taken not only from the present epidemy but from former ones, proving the above assertions.

I am happy to give your readers the conclusions of so scientific a body in regard to the good effects of vaccination, as I am well aware that, of late, the contrary opinion is gaining ground with a certain part of the population of England and the United States.

Let it be fully understood that the above statements have reference to vaccination that has taken effect anterior to an attack of small-pox, because the Commission are in accordance with good authority, when they declare that a person affected simultaneously, or nearly so, with small-pox and the eruption of vaccination, that both affections march independently the one of the other. In such a case the small-pox will take its natural course, either benign or malignant, as the case may be.

They also declare their disbelief in regard to vaccination being capable of attenuating the natural march of small-pox, when made at the beginning of this latter disease; yet, as popular opinion claims the contrary, they say there is no harm in vaccinating the person under the influence of variola, and if the patient demands it, the shrewd physician will give him this consolation. It will be perceived that the Paris Commission do not admit the doctrine of celebrated English authority—Aitken, for example—where he says, "a person exposed to the variolous contagion, if then vaccinated, it will mitigate the disease." The period of incubation has been, in general, from twelve to fourteen days.

Among the complications the most worthy to be noted are several cases of Endocarditis, Endopericarditis, Pericarditis, softening of the heart, which occurred always in severe cases, no cardiac complication being noted in varioloid patients. Owing to the medical constitution now existing in Paris, the most frequent complications have been laryngeal, bronchial and pulmonary. In fact, we may say there is a sort of atmospherical epidemy now reigning in Paris, which attacks the tonsils of a great number of persons, lasting three, four and five days, causing great prostration and severe lumbar pains. With this latter I was affected, and had also to treat some of my friends for the same.

In regard to the treatment of variola, the attention of the Paris medical profession has principally been drawn to the medicament proposed by Dr. Chauffard, which consists in the employment of phenic acid in large doses; not as a specific, thus suppressing well-known indications of treatment, but as specially destined to act upon the pyrogenic and toxic accidents of the secondary fever.

The dose employed has been from 25 centigrammes to $1\frac{1}{4}$ grammes a-day. This medicament has been perfectly tolerated by the patients, causing no vomiting and only a little diarrhœa at the end of six or seven days' administration. It is not certain even that the diarrhœa was caused by this acid. Consecutive abscesses have been much less common where phenic acid has been given.

The patients have been daily washed with water containing this acid, and afterwards covered with powder from head to foot; linen often changed; windows often open, to give continually pure air; and whenever the rooms appeared vitiated, fumigations of juniper were employed.

At the same time the patients have had broth and some wine, from the beginning, and food as soon as they requested it.

In my next letter I propose to treat of a certain kind of tumor affecting the urinary meatus of women, a very interesting case just having been treated by myself and a good Paris surgeon.

A. W. BOSWORTH.

